# Radar Target Detection Algorithm Using Convolutional Neural Network to Process Graphically Expressed Range Time Series Signals

**DOI:** 10.3390/s22186868

**Published:** 2022-09-11

**Authors:** Yan Dai, Dan Liu, Qingrong Hu, Xiaoli Yu

**Affiliations:** 1 Beijing Institute of Radio Measurement, Beijing 100854, China; 2 Defense Technology Academy of China Aerospace Science and Industry Corporation Limited, Beijing 100070, China; 3 Beijing Research Institute of Telemetry, Beijing 100094, China

**Keywords:** low signal-to-noise ratio, convolutional neural network, graphical, detection probability

## Abstract

Under the condition of low signal-to-noise ratio, the target detection performance of radar decreases, which seriously affects the tracking and recognition for the long-range small targets. To solve it, this paper proposes a target detection algorithm using convolutional neural network to process graphically expressed range time series signals. First, the two-dimensional echo signal was processed graphically. Second, the graphical echo signal was detected by the improved convolutional neural network. The simulation results under the condition of low signal-to-noise ratio show that, compared with the multi-pulse accumulation detection method, the detection method based on convolutional neural network proposed in this paper has a higher target detection probability, which reflects the effectiveness of the method proposed in this paper.

## 1. Introduction

In the process of detecting long-range small targets, radar has the problem of low detection probability caused by the low signal-to-noise ratio (SNR), which affects the tracking and recognition. Traditional target detection methods use the amplitude information of echo signal to detect, but in the case of low SNR, the amplitude information is submerged in the noise, which is not conducive to target detection [1]. How to obtain a higher detection probability under the condition of low SNR is one of the research trends in the aspect of target detection. In this paper, more information of the echo signal is fully utilized through graphical expression, and some potential features that are unknown are automatically extracted from the graph through convolutional network, which may better reflect the characteristics of the target echo, so as to achieve better detection effect under the condition of low SNR.

At present, target detection methods for low SNR scenarios mainly include the coherent integration detection method [2,3,4,5,6], dynamic programming (DP) algorithm [7,8] greedy algorithm [9], and others [10,11]. The coherent integration method based on frequency distance offset correction was proposed in [2], it needs a search around the preset target speed. References [3,4] focus on correcting the target range travel based on Keystone transform, so as to improve the SNR during phase-coherent integration, but it is mainly used for the high-speed small targets. References [5,6] introduce the Track-Before-Detect (TBD) into radar target detection, mainly through the trajectory characteristics of the target to carry out the non-coherent accumulation between pulses. The improved DP algorithm proposed in [7] needs to predict the target position and assume that multiple target speeds should be extrapolated. The target velocity in the improved DP algorithm proposed in [8] is generally extrapolated from the historical trajectory of the target. The greedy algorithm proposed in the literature [9] is oriented to the classical point target detection and tracking field, but the algorithm requires a large amount of computation and needs accelerating. The traditional target detection only considers the amplitude of the echo signal, but there is much information in the pulse shape change in the target echo which is not used in the target detection. In a word, few existing methods can meet the stability and real-time requirements of radar target detection in low SNR scenarios.

In the existing wideband radar system, the narrow- and wideband transmission mode is generally adopted. Narrowband signals are used for target detection and tracking, and wideband signals are used for target recognition and imaging [12]. In this working mode, the imaging data rate of broadband is low, but the data rate has an impact on wideband imaging, and the imaging requires a larger data rate. Therefore, the research of target detection in wideband is beneficial to improve the data rate of wideband signal and improve the quality of feature acquisition. In the case of single scatterer, the SNR of point target echo under narrowband conditions is equivalent to that under wideband conditions. Therefore, the detection of a single scattering point is independent of the bandwidth [13,14]. In the case of more than one scatterer or distributed target, the detection of wideband signal has potential advantages compared with that of narrowband [15,16]. Under the condition of high SNR, wideband detection rate is better than narrowband detection rate [17]. Therefore, the research of this paper chooses the signal detection method under the condition of wideband.

Compared with traditional target detection methods, deep learning has shown a superior performance in target detection [18,19]. Compared with the previous manual extraction of target data features through experience, deep learning algorithms can automatically extract some unknown deep data features, thereby improving the effectiveness of feature extraction. Commonly used deep learning models include restricted Boltzmann machine (RBM) [20], auto encode (AE) [21], and convolutional neural network (CNN) [22]. Among them, CNN has great advantages in image recognition and target detection [23]. Compared with the other two learning models, CNN can extract more features, and the ability of feature expression is stronger; moreover, it can also realize feature extraction, feature selection, and feature classification at the same time which enhances the separability of features [24,25,26]. Convolutional neural network has excellent feature extraction ability, and can extract higher level semantic features in the image without feature engineering, and has superior image classification performance [27]. This ability has attracted more and more attention in target detection. Therefore, CNN is adopted in this paper to complete the learning of target features, thereby improving the detection probability of the target in the case of low SNR.

The rest of this paper is structured as follows: Section 2 analyzes the graphical expression of echo signal, while Section 3 designs the radar target detection network, five simulation experiments are presented to verify the effectiveness of proposed target detection method in Section 4, and finally Section 5 concludes the article.

## 2. Graphical Expression Signal

### 2.1. Signal Definition and Matched Filtering

The linear frequency modulation signal (LFM) transmit waveform can be expressed as:

(1) $$s_{0}\left(t\right)=\mathrm{rect}\left(\frac{t}{T}\right)A\exp\left(j\pi kt^{2}+j2\pi f_{0}t\right)$$

where $\mathrm{rect}\left(\frac{t}{T}\right)=\left\{\begin{array}{cc} 1, & -T/2\leq t\leq T/2\\ 0, & others \end{array}\right.$ is the rectangular window function, T is the pulse width, A is the amplitude, $f_{0}$ is the carrier frequency, and k is frequency modulation slope.

The output signal of the chirp signal after matched filtering is:

(2) $$s_{t}\left(t\right)=\mathrm{rect}\left(\frac{t}{2T}\right)\left(T-\left|t\right|\right)\cdot \mathrm{sinc}\left(\pi kt\right)\exp\left(j2\pi f_{0}t\right)$$

the output signal has the envelope shape of the sinc function.

### 2.2. Two-Dimensional Echo Sequence Diagram of Multiple Cycles

The signal transmitted by the radar is set as a linear frequency modulated signal, and the received echo signal contains Gaussian white noise. After matching and filtering the received echo signal, the echo signal of each frame is obtained, which is the one-dimensional echo range image. The horizontal coordinate of the one-dimensional echo image is set as the range, and the vertical coordinate is set as the amplitude.

Assume that the transmitted chirp signal is:

(3) $$s_{1}\left(t\right)=A\exp\left(j\pi kt^{2}\right)$$

using the function to generate Gaussian white noise snv, the echo signal is:

(4) $$s_{t}\left(t\right)=s_{1}\left(t\right)+snv$$

a single frame one-dimensional echo signal is obtained by matched filtering, and by changing the distribution of Gaussian white noise in each echo signal, multi-frame echo signals with the same SNR can be obtained.

**Figure 1:**
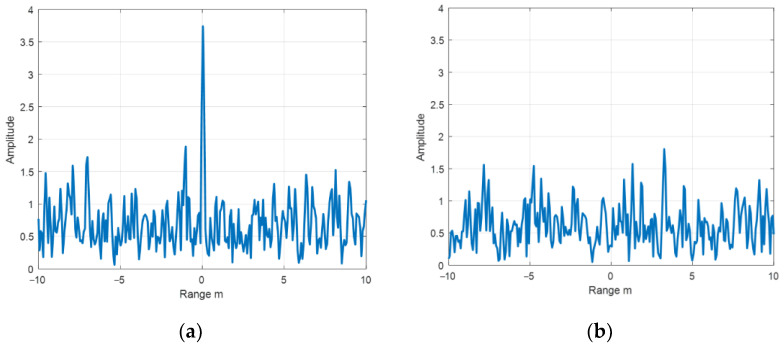
Echo diagrams of chirps with (**a**) SNR of 12 dB and (**b**) SNR of 0 dB.

The echo diagram of the chirp signal after matched filtering with different SNR is shown in Figure 1.

In the simulation experiment, the amplitude, bandwidth, pulse width, and frequency modulation slope of the transmitted signal are the same, and the distribution of Gaussian white noise in each frame is changed under the condition that the SNR of the echo remains unchanged. Under the condition of the same SNR, echo signals of multiple cycles can be collected to form a two-dimensional echo sequence diagram, the abscissa of the two-dimensional echo sequence diagram is the range unit, and the ordinate is the echo cycle. The SNR of the echo signal can be changed by changing the amplitude of the transmitted signal and the power of the noise, so that two-dimensional echo sequence diagrams under different SNR can be obtained.

**Figure 2:**
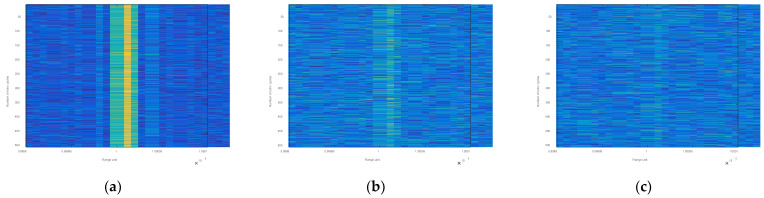
Two-dimensional echo sequence diagrams of 500 echo cycles under different SNR of (**a**) 12 dB, (**b**) 0 dB, (**c**) −5 dB.

In this paper, a multi-period two-dimensional echo sequence is used as the input data of target detection. The two-dimensional echo sequence diagrams of 500 echo cycles under different SNR are shown in Figure 2.

## 3. Design of Radar Target Detection Network

Radar target detection Network is designed based on Convolution Neural Network. It is a special multi-layer perceptron, which can realize target recognition by extracting various features of original data and then learning according to the features.

### 3.1. Structure and Parameters of Convolutional Neural Network

**Figure 3:**
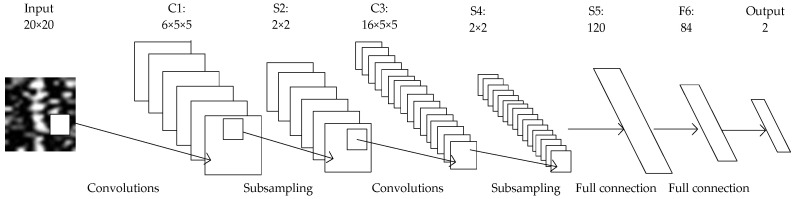
The structure diagram of the convolutional neural network in this paper.

Convolutional neural network consists of input layer, convolutional layer, pooling layer, fully connected layer, and output layer. In this paper, the convolutional neural network is improved on the classical convolutional neural network Lenet5 [19]. Figure 3 is the structure diagram of the convolutional neural network in this paper.

**Figure 4:**
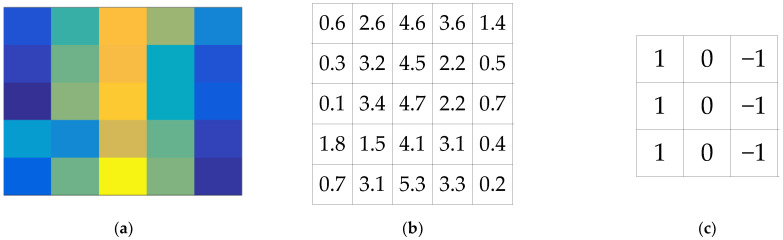
Convolutional neural network (**a**) input image, (**b**) amplitude value, and (**c**) convolution kernel.

**Figure 5:**
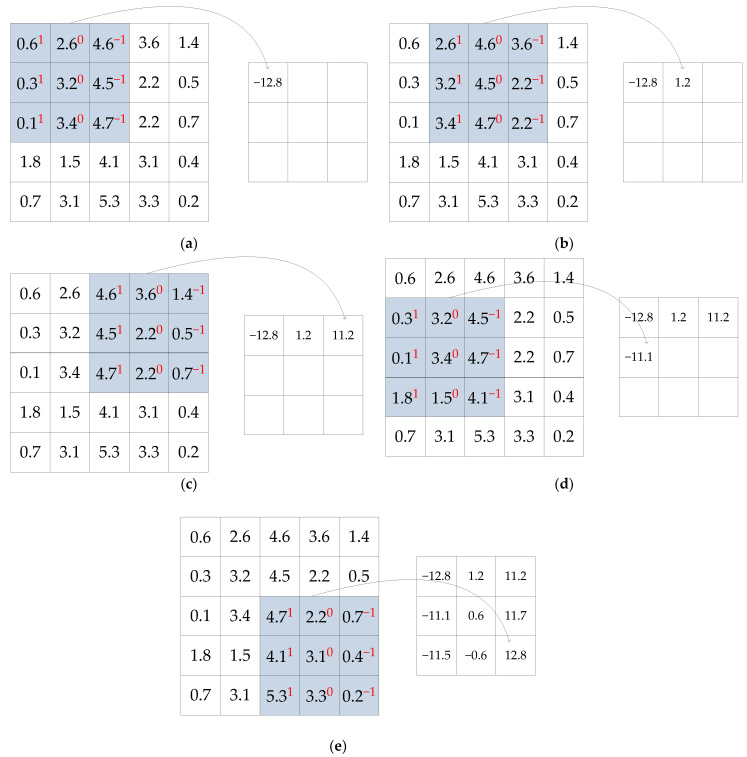
Convolution process with the result of (**a**) the first convolution, (**b**) the second convolution, (**c**) the third convolution, (**d**) the fourth convolution, and (**e**) the last convolution.

1. Input layer: the input layer is used to receive the original input data or images and preprocess them.
2. Convolutional layer: The convolutional layer is the core of the convolutional neural network, which acts on the information features of data or images through a certain size of convolution kernel. The main function of the convolutional layer is to extract features, and different features are calculated by different convolution kernels [24]. Assume that the input image is a 5 × 5 matrix, and the radar simulation echo signal image of 12 dB SNR is taken as the input image, as shown in Figure 4a, the amplitude value is shown in Figure 4b, and the convolution kernel is a 3 × 3 matrix as shown in Figure 4c:

The first element of the convolutional neural network is in the upper left corner in Figure 4b; in the first step, the convolution kernel of Figure 4c is used, it is overlayed on the input image, as shown in Figure 5a, and then the element multiplication operation is performed. Finally, the first convolution result element is obtained by adding each element of the matrix. Then the convolution operation is performed in turn, and the last convolution result as shown in Figure 5e.

The convolution kernel used in Figure 4b is a vertical edge detector, in addition, there are horizontal edge detector, Sobel filter, Scharr filter, and so on. The nine numbers of the convolution kernel are regarded as nine parameters, and the parameter values are set to detect features.

In the convolution process shown in Figure 5, the convolution step is set to 1, that is, it moves one grid at a time. If the step size is set to 2, it moves two grids each time.

Assuming that the input image is n×n, the convolution kernel is f×f, and the step is s, the size of the output image after one convolution is:

(5) $$\left(\frac{n-f}{s}+1\right)\times \left(\frac{n-f}{s}+1\right)$$

3. Pooling layer: Pooling layer is a “down-sampling” operation, which filters out the minor features in the data and retains the important feature information. Max pooling or average pooling is usually used.

**Figure 6:**
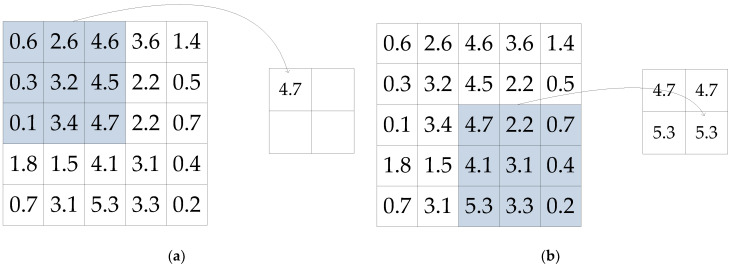
Pooling process with result of (**a**) the first pooling and (**b**) the last pooling.

The function of the max pooling operation is that as long as a feature is extracted in any quadrant, it remains in the output of the max pooling. For the input image in Figure 4a, the max pooling process and the result are shown in Figure 6 with a 3 × 3 pooling layer and a sliding step size of 2.

Equation (5) used to calculate the output size of the convolution layer is also applicable to calculate the max pooling output size.

4. Fully connected layer: After the processing of the convolution layer and the pooling layer, one or two fully connected layers are generally added before the output layer. Each node in the fully connected layer is connected to the nodes in the previous layer, and the previously extracted features are integrated through the fully connected layer to complete the prediction or classification task.
5. Output layer: the output layer is the final result generation layer.

The parameters of the convolutional neural network mainly include: the number of neural network layers, the size of the convolution kernel and the selection of the sliding step, the activation function, the number of training times, the size of the pooling layer, and so on.

### 3.2. Target Detection Network Design

Convolutional neural networks were successfully applied in target detection in 1994 [28]. For a long time, the application of convolutional neural networks in target detection has not made progress due to the lack of data, device constraints, and over-fitting. In 2012, AlexNet made a major breakthrough in image recognition [29]. Since then, the application of convolutional neural network in target detection has received more and more attention and research [30,31,32,33,34]. Target detection based on convolutional neural network has indeed made some progress, and has certain advantages over traditional target detection methods.

LeNet5 is a classical convolutional neural network, and researchers have proposed new CNN models based on it. It mainly includes: AlexNet, a large deep convolutional neural network proposed in 2012; in 2014, VGGNet with regular network structure was proposed [35]; in 2014, InceptionNet using different sizes of kernels and batch normalization in one layer was proposed [36]; and in 2015, ResNet, which uses residual hopping between layers to introduce forward information, was proposed to make it possible to deepen the number of layers in neural networks [37].

**Figure 7:**
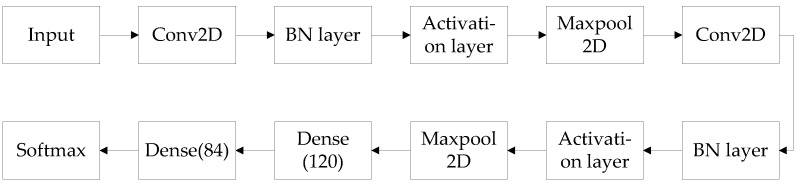
The frame diagram of the convolutional neural network in this paper.

Compared with other networks, LeNet5 has the smallest input image size, number of network layers, number of weight parameters, and number of product operations. Target detection should not blindly pursue network depth. Although many different structure forms have been developed, the basic structure of neural networks for target signal detection includes convolutional layer, pooling layer, and fully connected layer. Therefore, the convolutional neural network built in this paper is improved on the basis of LeNet5 structure, so that better target detection results can be obtained under the condition of smaller image size and fewer network layers. Figure 7 is the frame diagram of convolutional neural network in this paper, and Table 1 is the network parameters.

In the future, the application and improvement of other convolutional neural networks in target detection will be further studied according to different target detection problems.

**Figure 8:**
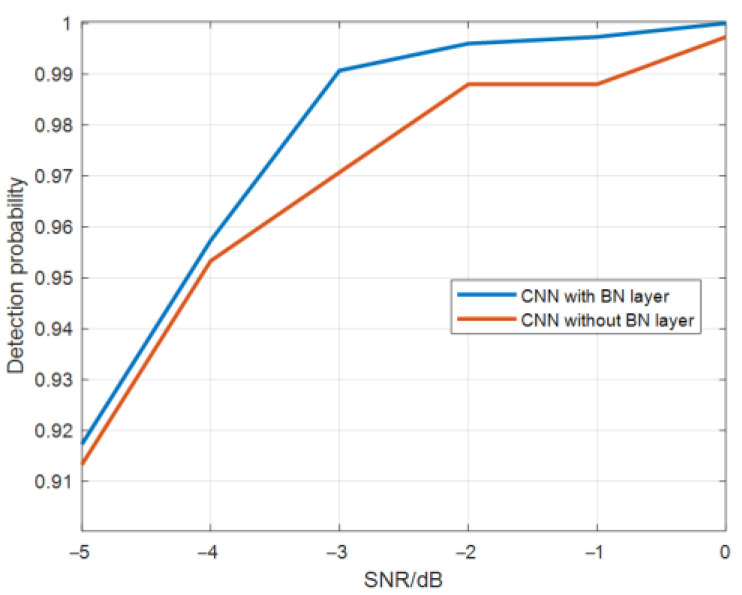
Target detection probability comparison graph of CNN with and without BN layer.

Compared with the structure of LeNet5, it can be seen that the convolutional neural network used in this paper adds two batch normalization (BN) layers, which normalizes a small batch of data, which can pull the original offset feature data back to 0 mean value, then the data entering the activation function is distributed in the linear region of the activation function. The small changes in the input function are more clearly reflected in the output of the activation function, and the discrimination power of the activation function on the input data are improved. Figure 8 is the comparison diagram of CNN target detection probability with and without a BN layer.

As can be seen from the Figure 8, the detection probability with a BN layer is slightly higher than that without a BN layer.

The output layer softmax has two neurons. This paper is a two-class problem: It detects whether there is a target in the input image. If there is a target, the output is 1, and if there is no target, the output is 0.

According to the parameters of the convolutional neural network set in this article, the input image is n×n, the convolution kernel size is $f_{1}=5$, the convolution step size is $s_{1}=1$, the pooling layer size is $f_{2}=2$, the pooling step size is $s_{2}=2$, and the output after the convolution layer and the pooling layer should be at least 2 × 2. To characterize the image features, according to Equation (5), the following can be found:

(6) $$\frac{\frac{\frac{\frac{n-f_{1}}{s_{1}}+1-f_{2}}{s_{2}}+1-f_{1}}{s_{1}}+1-f_{2}}{s_{2}}+1\geq 2,n\geq 20$$

According to the solution result of Equation (6), it can be seen that the size of the input image should be at least 20 × 20, and the output result can represent the data features after being processed by two convolution layers and two pooling layers of convolutional neural network. Otherwise, when the input image size is too small, the output is less than two element values after the convolutional neural network processing, and the data features cannot be characterized.

**Figure 9:**
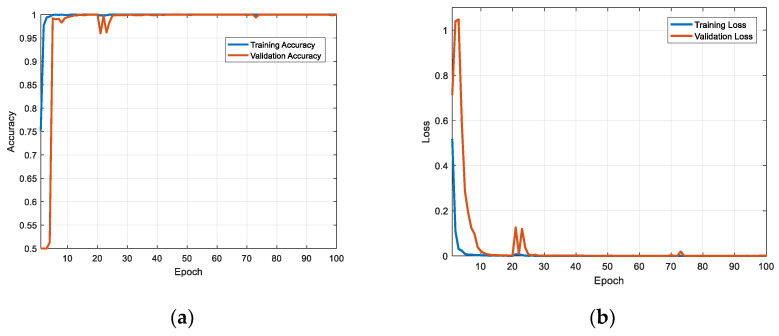
(**a**) The training/verification accuracy curve and (**b**) the training/verification loss curve of image block 20 × 20 in the case of 0 dB SNR.

Concerning the CNN, it is customary to show the training/validation versus epoch curves. That provides an idea of the quality of the training and the generalization ability of the trained neural network. Figure 9 is the training/verification accuracy curve and the training/verification loss curve of image block 20 × 20 in the case of 0 dB SNR.

As can be seen from Figure 9, with the increase in training times, the accuracy and loss value tend to be stable. The accuracy tends to 1 and the loss tends to 0. This verified that the quality and generalization ability of the trained convolutional neural network were relatively good.

## 4. Experiments and Discussions

In this section, five simulation experiments are presented to verify the effectiveness of the proposed target detection method. Firstly, in order to verify that the effect of the method is not caused by the gain of multi-period pulse accumulation, the convolutional neural network method is compared with the multi-pulse accumulation detection method, the convolutional neural network method is validated to detect a single scattering point with low SNR. Secondly, the influence of a two-dimensional image block size on false alarm probability and detection probability of target detection is studied by changing the cycle number and range number of image block. Thirdly, the convolutional neural network is used to detect a single moving scattering point under the condition of SNR from 8 dB to 12 dB, which verifies that the convolutional neural network has a good detection effect on the scattering point with velocity at a lower SNR. Fourthly, the training set of a single scattering point is used to detect two scattering points at different distances to verify the universality of the proposed method. Fifthly, the multi-scatterer target is detected under the condition of wideband and narrowband, and the target detection under the condition of wideband and narrowband is compared and analyzed, and the effectiveness of the proposed method for multi-scatterer target detection is verified.

**Figure 10:**
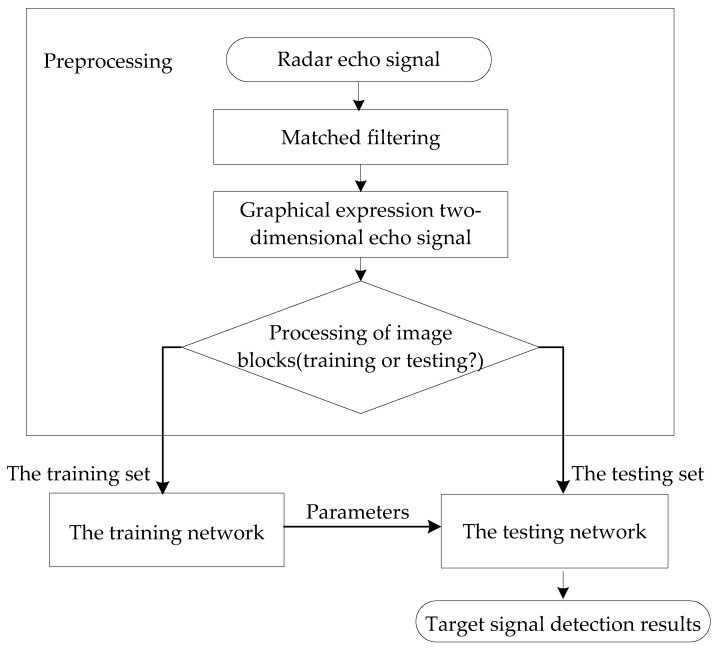
The flowchart of radar target signal detection in this paper.

The flowchart of the radar target signal detection in this paper is shown in the Figure 10.

The simulation parameters of LFM are shown in the Table 2.

### 4.1. Processing of Image Blocks

For the 500 × 20,000 two-dimensional echo sequence diagram, 500 is the number of echo cycles, and 20,000 is the echo distance unit, then the distance unit of the target existence is 10,001. The input image of the convolutional neural network is a gray scale image, and the color depth represents the energy. If there is a target in the echo, the higher the target energy value, the lighter its color.

**Figure 11:**
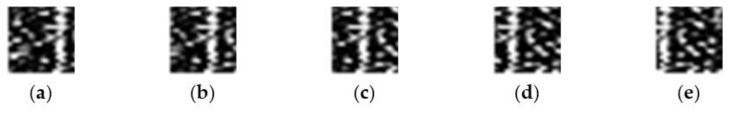
Partial 20 × 20 sample graph with target under the condition of 0 dB SNR, and the target in unit positions are (**a**) 0.8, (**b**) 0.65, (**c**) 0.5, (**d**) 0.3, and (**e**) 0.15.

**Figure 12:**
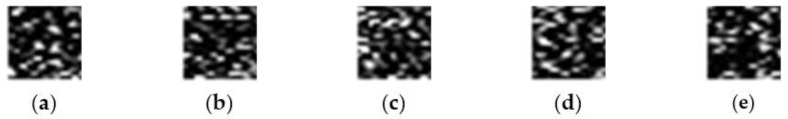
Partial 20 × 20 sample graph without target under the condition of 0 dB SNR, and the units are (**a**) 1000th, (**b**) 2000th, (**c**) 3000th, (**d**) 4500th, and (**e**) 6000th.

For 20 × 20 image blocks: move the echo range unit by 5, 8, 11, 15, and 18, then the range unit where the target is located becomes 9996, 9993, 9990, 9986, and 9983, the blocks are divided by 20, then the range block positions where the target is located are 4999.8, 4999.65, 4999.5, 4999.3, and 4999.15, the targets are all on the 5000th range block unit, but at different positions of the unit: 0.8, 0.65, 0.5, 0.3, and 0.15, respectively. Figure 11 is a graph of target samples at different positions when the SNR is 0 dB; the non-target samples are taken from the 1000th, 2000th, 3000th, 4500th, and 6000th range block units, Figure 12 is a graph of non-target samples at different positions when the SNR is 0 dB.

**Figure 13:**
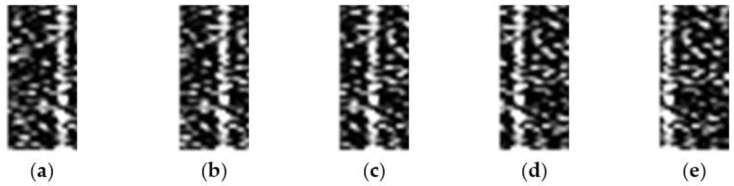
Partial 20 × 40 sample graph with target under the condition of 0 dB SNR, and the target in unit positions are (**a**) 0.8, (**b**) 0.65, (**c**) 0.5, (**d**) 0.3, and (**e**) 0.15.

**Figure 14:**
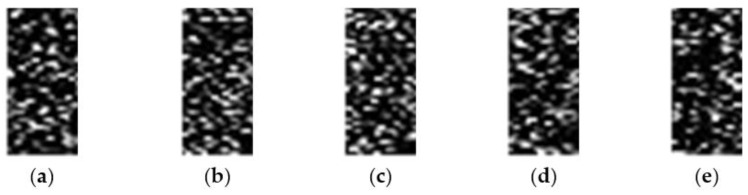
Partial 20 × 40 sample graph without target under the condition of 0 dB SNR, and the units are (**a**) 1000th, (**b**) 2000th, (**c**) 3000th, (**d**) 4500th, and (**e**) 6000th.

For 20 × 40 image blocks (20 is the number of distance units, 40 is the number of echo cycles): the value positions of the target and the value positions of non-target are the same as the value position of the 20 × 20 image blocks. Figure 13 is a graph of target samples at different positions when the signal-to-noise ratio is 0 dB; Figure 14 is a graph of non-target samples at different positions when the signal-to-noise ratio is 0 dB.

**Figure 15:**
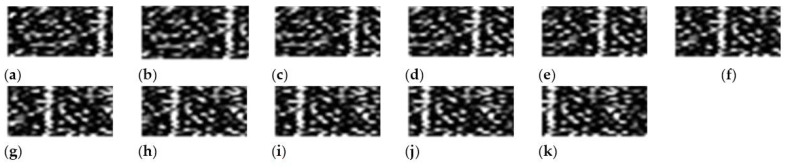
Partial 40 × 20 sample graph with target under the condition of 0 dB SNR, and the target in unit positions are (**a**) 0.9, (**b**) 0.825, (**c**) 0.75, (**d**) 0.65, (**e**) 0.575, (**f**) 0.45, (**g**) 0.4, (**h**) 0.325, (**i**) 0.25, (**j**) 0.175, and (**k**) 0.1.

**Figure 16:**
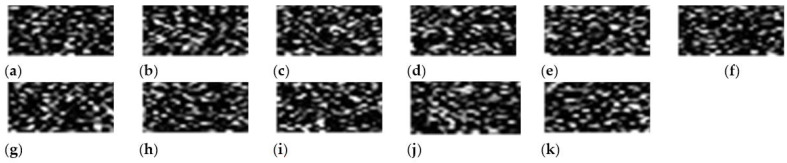
Partial 40 × 20 sample graph without target under the condition of 0 dB SNR, and the units are (**a**) 400th, (**b**) 800th, (**c**) 1200th, (**d**) 1600th, (**e**) 2000th, (**f**) 2400th, (**g**) 2800th, (**h**) 3200th, (**i**) 3600th, (**j**) 4000th, and (**k**) 4400th.

For 40 × 20 image blocks (40 is the number of distance units, 20 is the number of echo cycles): move the echo range unit by 5, 8, 11, 15, 18, 23, 25, 28, 31, 34, and 37, the blocks are divided by 40, and the targets are all on the 2500th range block unit, but at different positions of the unit: 0.9, 0.825, 0.75, 0.65, 0.575, 0.45, 0.4, 0.325, 0.25, 0.175, and 0.1, respectively. Figure 15 is a graph of target samples at different positions when the SNR is 0 dB; the non-target samples are taken from the 400th, 800th, 1200th, 1600th, 2000th, 2400th, 2800th, 3200th, 3600th, 4000th, and 4400th range block units, Figure 16 is a graph of non-target samples at different positions when the signal-to-noise ratio is 0 dB.

### 4.2. Validation Experiments of the Detection Method

#### 4.2.1. Experiment I: Validation of the Detection Method

**Figure 17:**
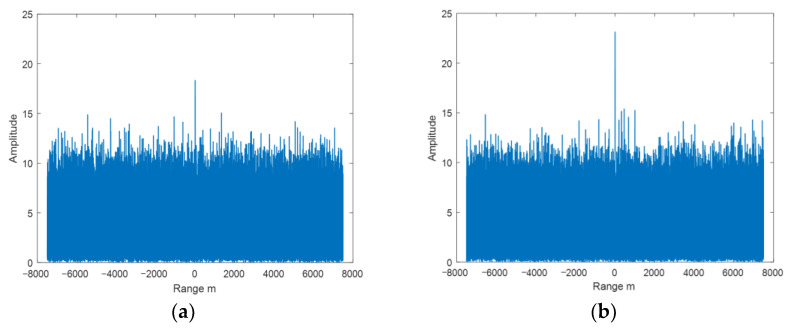

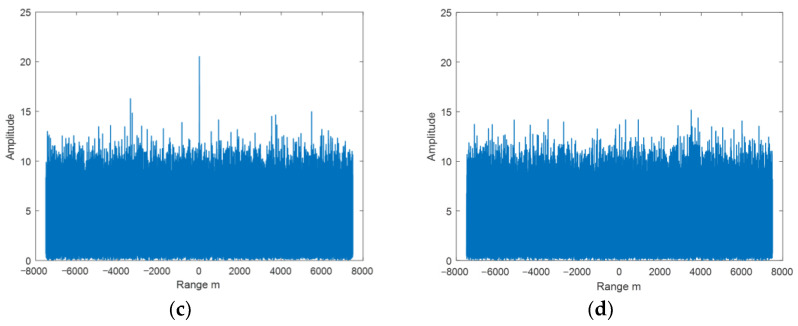
The echo diagram of 20 echo period signals with accumulated SNR of (**a**) 12.6 dB, (**b**) 14.6 dB, (**c**) 13.5 dB, and (**d**) 10.9 dB under the condition of 0 dB SNR.

Assume that the target is a single scattering point, satisfying the Swerling0 model. For the signal with 20 echo cycles, the theoretical power accumulation gain of the signal is about 26 dB, and the theoretical power accumulation gain of the noise is about 13 dB, so the theoretical SNR gain is 13 dB. In practice, the SNR of 20 echo cycles fluctuates due to echo fluctuation. Figure 17 shows the accumulated echo diagram of 20 echo period signals under the condition of 0 dB SNR, and Table 3 shows the power values of 20 echo period signals under the condition of 0 dB SNR.

According to the data in Table 3, the average value of the 8 SNRs is 13.1 dB. In the case of 0 dB SNR, the SNR fluctuates around 13 dB after 20 echo cycles of accumulation, and the average SNR after accumulation approaches about 13 dB with more simulation times.

For the two-dimensional target echo sequence diagram with 0 dB SNR, the accumulated SNR is about 13 dB when it is divided into 20 × 20 blocks. The sample number of convolutional neural network training set is 15,000, and the sample number of test set is 10,000.

The detection results of the test set are: the number of false alarm samples is 0 and the detection probability is 1.

For mono-pulse, linear detection, and non-undulating targets, the detection probability is about 0.98, when the false alarm probability is 1 × 10^−^^4^ and the SNR is 13 dB [38]. For the multi-pulse accumulation detection method under the condition of 0 dB SNR, the theoretical SNR is 13 dB after 20 echo cycles, and the detection probability is 0.9860 after 10,000 Monte Carlo simulations on the simulated echoes.

**Figure 18:**
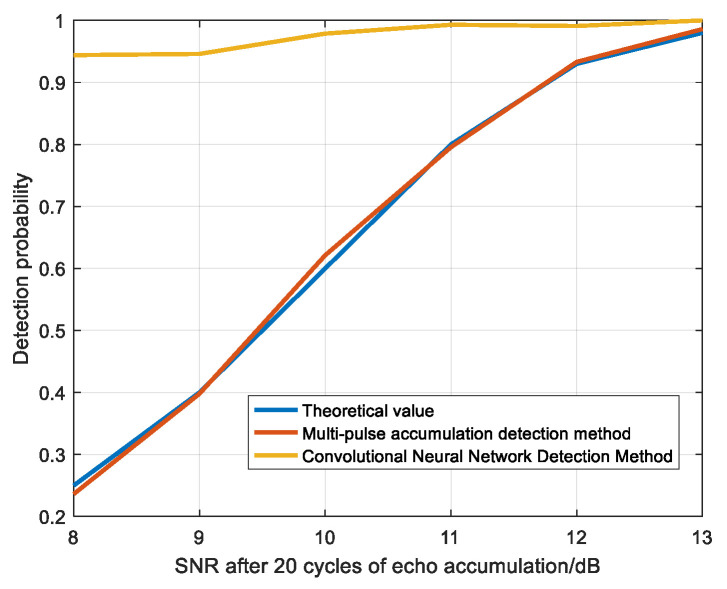
The comparison diagram of the detection probability between the multi-pulse accumulation detection method and the method in this paper.

The above experimental steps were repeated to obtain the detection probability of the multi-pulse accumulation detection method and the method in this paper under the conditions of 0 dB, −1 dB, −2 dB, −3 dB, −4 dB, and −5 dB SNR, as shown in Figure 18.

As can be seen from Figure 18, within the allowable error range, the detection probability of the multi-pulse accumulation detection method is basically consistent with the theoretical detection probability, which verifies the effectiveness of the simulation experiments in this paper. When the signal-to-noise ratio of the original echo signal is as low as −5 dB, the theoretical SNR of 20 echoes after accumulation is 8 dB, the detection probability of the convolutional neural network detection method is higher than 0.9, while the detection probability of the multi-pulse accumulation detection method is 0.2363. The detection probability of the proposed method is much better than that of the multi-pulse accumulation detection method in the case of low SNR, which verifies the effectiveness of the method in this paper.

#### 4.2.2. Experiment II: Influence of Image Block Size on Detection

**Figure 19:**
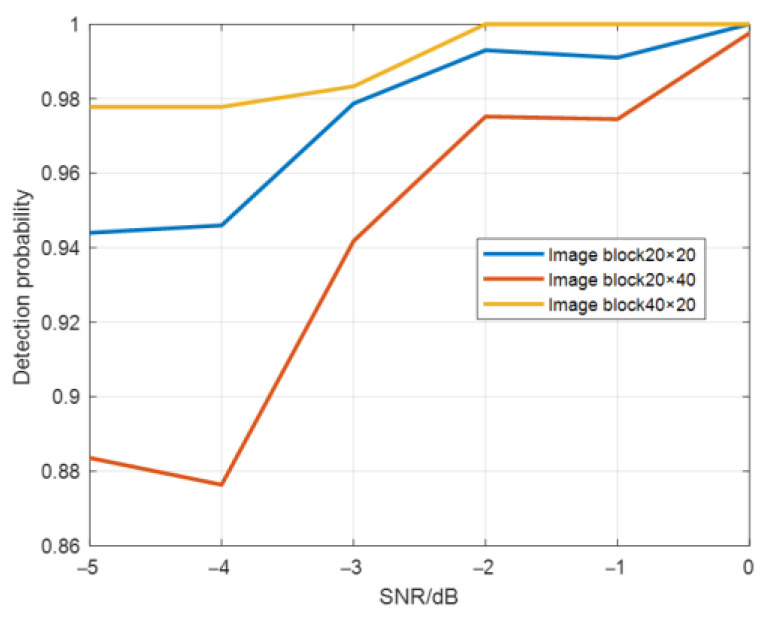
Target detection probability graph using CNN for different image block sizes.

**Figure 20:**
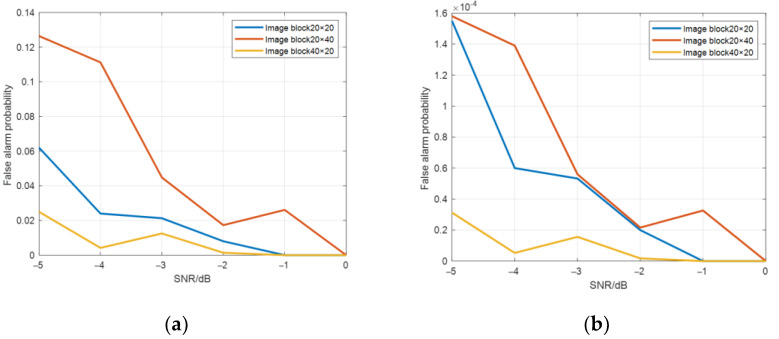
False alarm probability graphs of (**a**) the whole image and (**b**) a single point with different image block sizes.

The range number and cycle number of image blocks were changed and the size of image blocks were set to 20 × 20, 40 × 20, and 20 × 40 (range number × cycle number). The target detection probability is shown in Figure 19, and the false alarm probability is shown in Figure 20.

As can be seen from Figure 19, when the image block size is 20 × 20 and 40 × 20, the cycle number of the image block is the same, but the detection probability decreases as the range number increases. As the range number increases, the proportion of effective features of the image block decreases, thus the detection probability decreases. When the image block size is 20 × 20 and 20 × 40, the range number of the image block is the same, but the detection probability increases with the increase in the number of cycles. As the number of cycles increases, the proportion of the effective features of the image block increases, so the detection probability increases.

When the number of cycles changes from 20 to 40, the SNR gain for multi-period pulse accumulation is 3 dB, and the detection probability of the method in this paper does not have the gain effect of 3 dB. Therefore, the effect of the method in this paper is not caused by the gain of multi-period pulse accumulation.

As can be seen from Figure 20, the variation trend of false alarm probability is opposite to that of detection probability, and the false alarm probability of a single point is about 10^−^^4^ orders of magnitude.

#### 4.2.3. Experiment III: Detection of Moving Scattering Point

Assume that there is a moving scattering point in the echo signal and that it moves radially relative to the radar. The velocity v of the scattering point is a fixed value, and the initial distance between the scattering point and the radar is $R_{0}$. The transmitted signal is LFM, then the multi-period echo signal is:

(7) $$s_{r}\left(t\right)=\sum_{i=0}^{M-1}A\mathrm{rect}\left(\frac{\alpha t-\tau_{0}-iT_{r}}{T_{p}}\right)\exp\left(j\pi k{\left(\alpha t-\tau_{0}\right)}^{2}+j2\pi f_{0}\left(\alpha t-\tau_{0}\right)\right)$$

where A is the amplitude, M is the pulse number and *i* is the *i*th pulse, $\tau_{0}=2R_{0}/c$ is the initial echo delay between the scattering point and the radar, $T_{P}$ is the pulse width, $T_{r}$ is the pulse repetition time, *t* is the time component, fast time $t_{s}\in \left[-T_{p}/2,T_{p}/2\right]$, and slow time $T_{i}=iT_{r}$; where α=(c−v)/(c+v)≈1−2v/c is the scale modulation factor, then:

(8) $$\alpha t-\tau_{0}=\left(t-\tau_{i}\right)+(-2v/c)t_{s}$$

where fast time $\tau_{i}=\tau_{0}+\left(2v/c\right)\cdot iT_{r}$ is the double delay of the real-time distance corresponding to the *i*th pulse. When the scattering point velocity is small, the pulse width is limited, and the intra-pulse scale modulation effect is not obvious, the echo signal can be simplified as follows:

(9) $$s_{r}\left(t\right)=\sum_{i=0}^{M-1}A\mathrm{rect}\left(\frac{t_{s}-\tau_{i}}{T_{p}}\right)\exp\left(j\pi k{\left(t_{s}-\tau_{i}\right)}^{2}+j2\pi f_{0}\left(t_{s}-\tau_{i}\right)\right)$$

**Figure 21:**
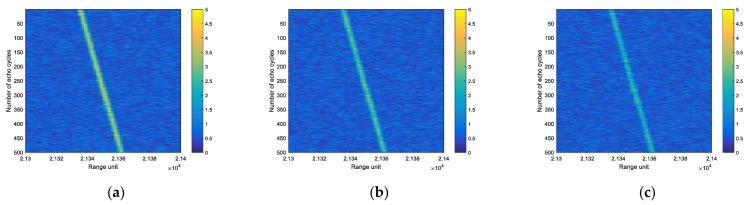
The echo diagrams of multi-period scattering point under different SNR of (**a**) 12 dB, (**b**) 10 dB, (**c**) 8 dB.

In the simulation experiment, the velocity v of the scattering point is 200 m/s, $T_{r}$ is 2 m/s, $R_{0}$ is 10 km and the other parameters are consistent with those in Table 2. Figure 21 shows the echo diagrams of a multi-period scattering point under different SNR.

**Figure 22:**
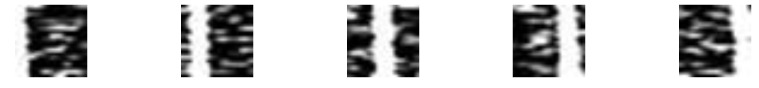
Partial 20 × 20 sample graph with target under the condition of 12 dB SNR.

**Figure 23:**
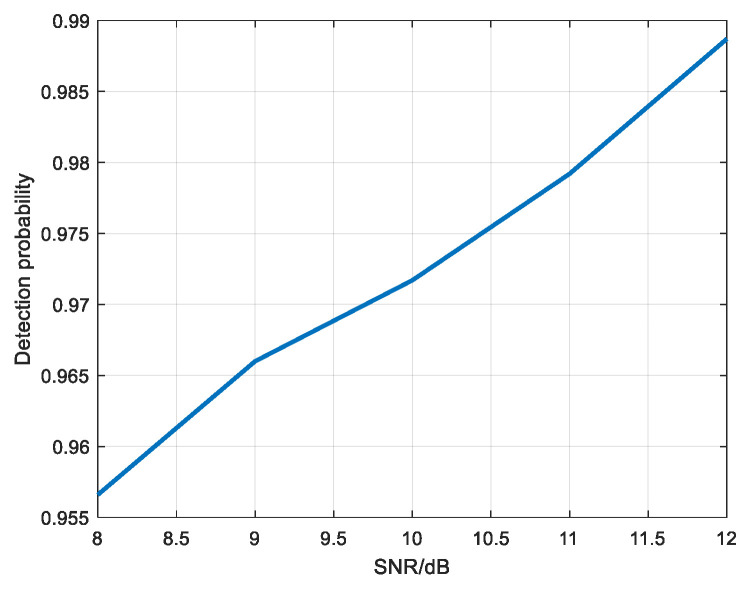
Detection probability diagram of scattering point under different SNR.

The convolutional neural network was used to detect the scattering point echo with SNR of 12 dB, 11 dB, 10 dB, 9 dB, and 8 dB. Figure 22 is the sample graph of target samples, which is the sample graph of the target at different positions in the picture. Figure 23 shows the detection probability of scattering points under different SNR.

As can be seen from Figure 23, when the SNR is 8 dB, the detection probability reaches above 0.95, so convolutional neural network has a good detection effect for moving the scattering point.

**Figure 24:**
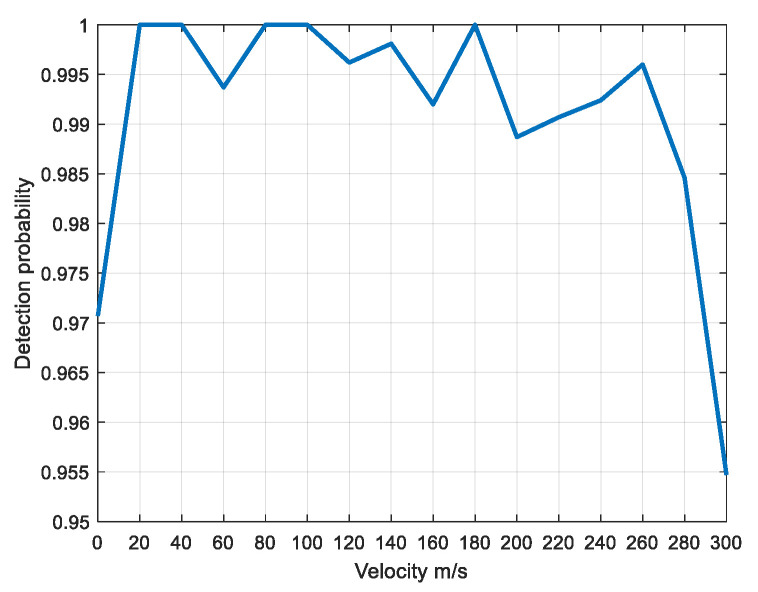
Detection probability diagram of scattering point under different speeds.

When the SNR is 12 dB, the scattering point echo samples with a speed of 200 m/s are the training set. Target echoes with a speed of 100 m/s to 300 m/s (with an interval of 20 m/s) are detected. Figure 24 shows the detection probability of different speeds.

As can be seen from Figure 24, when the speed is 20 m/s to 280 m/s, the detection probability is above 0.985. When the speed is 20 m/s, the detection probability is about 0.97, and when the speed is 300 m/s, the detection probability decreases to about 0.955. Therefore, for the echoes with a speed of 200 m/s as the training set, the target echoes with a speed of 20 m/s to 280 m/s have a good detection effect, which verifies the universality of the proposed method.

#### 4.2.4. Experiment IV: Detection of Two Scattering Points

Suppose there are two scattering points in the echo signal, and the radial distance of the two scatterers relative to the radar is $r_{1}$, then the echo signal is:

(10) $$\begin{array}{cc} s\left(t\right) & =\mathrm{rect}\left(\frac{t}{T}\right)A_{1}\exp\left(j\pi kt^{2}+j2\pi f_{0}t\right)+\\ & \mathrm{rect}\left(\frac{t-t_{1}}{T}\right)A_{2}\exp\left(j\pi k{\left(t-t_{1}\right)}^{2}+j2\pi f_{0}(t-t_{1})\right) \end{array}$$

where $A_{1}$ is the amplitude of scattering point 1, $A_{2}$ is the amplitude of scattering point 2, and $t_{1}=\frac{2r_{1}}{c}$ is the echo delay between scattering point 2 and scattering point 1.

According to the simulation parameter values in Table 2, the range resolution can be calculated as:

(11) $$\Delta R=\frac{c}{2B}=0.15m$$

**Figure 25:**
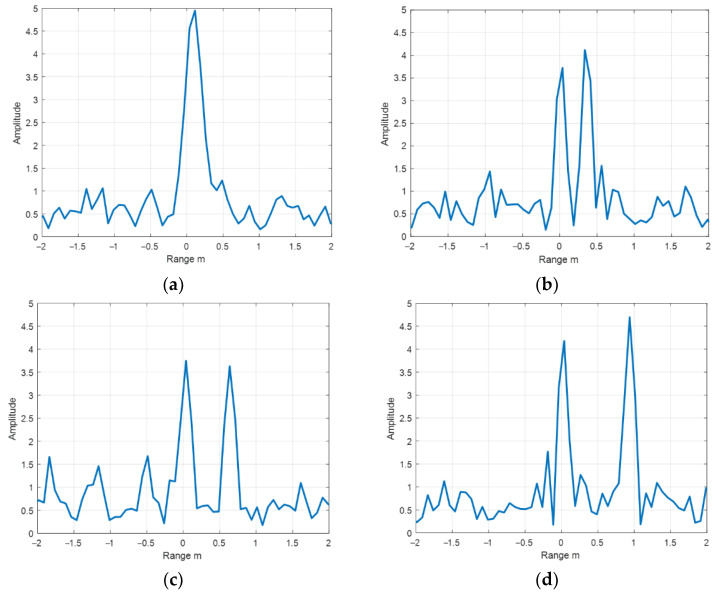
The echo diagram of two scattering points with a distance of (**a**) 0.15 m, (**b**) 0.3 m, (**c**) 0.6 m, and (**d**) 0.9 m under the condition of 12 dB SNR.

The distance of the two scattering points were changed to 0.15 m, 0.3 m, 0.6 m, and 0.9 m. Figure 25 is the echo diagram of two scattering points with SNR of 12 dB at different positions.

As can be seen from Figure 25 when the distance between two scattering points is 0.15 m, two scattering points cannot be distinguished in the signal echo diagram, and only one spectral peak can be distinguished in the echo diagram, which can be approximated to the echo of a single scattering point. When the distance between two scattering points is 0.3 m, two spectral peaks can be distinguished in the signal echo diagram, but the echo of the two scattering points cannot be completely separated, and the echo becomes uncertain with the change in noise. When the distance between the two scattering points is 0.6 m, two spectral peaks can be distinguished in the signal echo diagram, and the echo of the two scattering points can be clearly distinguished, but the echo of the two scattering points has a small amount of folding. When the distance between the two scattering points is 0.9 m, two spectral peaks can be distinguished in the signal echo diagram, the echo of the two scattering points can be more clearly distinguished, and the echo of the two scattering points has little influence on each other.

**Figure 26:**
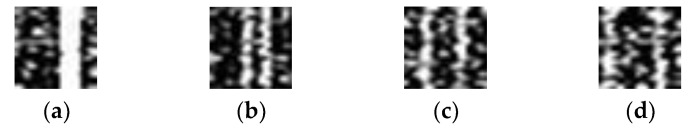
Partial 20 × 20 sample graph with target under the condition of 0 dB SNR, and the two scattering points with a distance of (**a**) 0.15 m, (**b**) 0.3 m, (**c**) 0.6 m, and (**d**) 0.9 m.

**Figure 27:**
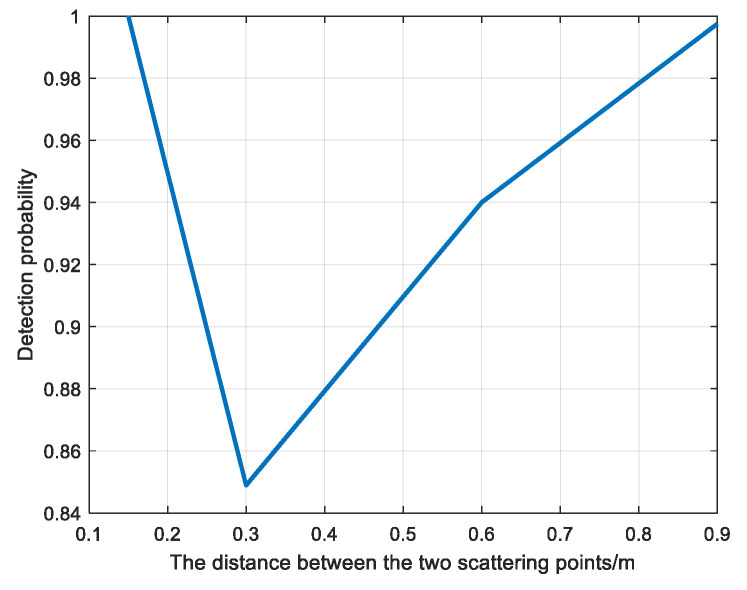
Detection probability diagram of two scattering points at different distances.

The convolution neural network was used to detect the echoes of two scattering points at different distances when the SNR was 0 dB, and the training set was a single scattering point echo sample at 0 dB in Section 4.2. Figure 26 is the sample graph of target samples at different distances, and Figure 27 is the detection probability diagram of two scattering points at different distances.

As can be seen from Figure 26, when the distance between two scattering points is 0.15 m, the two scattering points cannot be distinguished, and their echo energy is higher than that of a single scattering point. When the distance between the two scattering points is 0.3 m, the two scattering points can be distinguished, but not completely. When the distance between the two scattering points is 0.6 m, the two scattering points can be distinguished. When the distance between the two scattering points is 0.9 m, the two scattering points can be completely distinguished.

The training set of convolutional neural network is the echo sample of a single scattering point, so the echo feature extracted is the echo feature of a single scattering point. As can be seen from Figure 27, when the distance between the two scattering points is 0.3 m, the echo characteristics of the two scattering points are inconsistent with those of a single scattering point, so the detection probability is the lowest. When the distance between the two scattering points is 0.15 m, the echo characteristics of the two scattering points are consistent with those of a single scattering point because the echoes of the two scattering points cannot be distinguished, and the echo energy is accumulated, so the detection probability is 1 at the highest. When the distance between the two scattering points is 0.6 m and 0.9 m, the echo characteristics of the two scattering points are basically consistent with those of the single scattering point because the echo characteristics of the two scattering points can be distinguished, and the detection probability is above 0.9. However, two scattering points with a distance of 0.9 m are more distinguishable than those with a distance of 0.6 m. Therefore, the detection probability of two scattering points with a distance of 0.9 m is slightly higher than that of two scattering points with a distance of 0.6 m.

### 4.3. Experiment V: Detection of Multi-Scatterer Target

In the case that the target has more than one scattering point, some scattering points cannot be distinguished in the narrow band because of the low range resolution. In this case, the echo amplitude of scattering points in narrowband is the vector synthesis of each scattering point. When the phase of each scattering point is different, the echo amplitude changes along with it. Therefore, an electromagnetic simulation model is built in this section, which is an aircraft model with 27 scattering points. The initial phase of the scattering points is changed by rotating the angle of the model. Two electromagnetic simulation experiments are built in this section to conduct a comparative analysis of wideband and narrowband detection, and verify the detection method in this paper.

#### 4.3.1. Electromagnetic Simulation Experiment I

**Figure 28:**
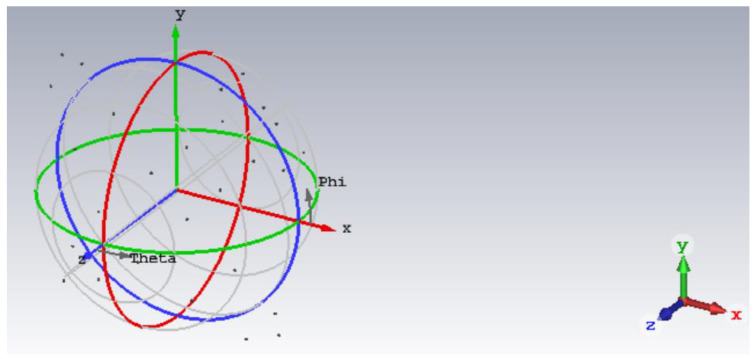
Electromagnetic simulation Experiment I model diagram.

A total of 27 metallic spheres with a radius of 10 mm were set, the model is shown in Figure 28. The software of the electromagnetic simulation experiment is CST, the method of solving is PO + PTD. Table 4 shows the coordinate positions of 27 metallic spheres. Electromagnetic simulation was carried out for the echoes in the case of wideband and narrowband. The frequency is [9.5, 10.5] GHz in the case of wideband and [9.995, 10.005] GHz in the case of narrowband.

**Figure 29:**
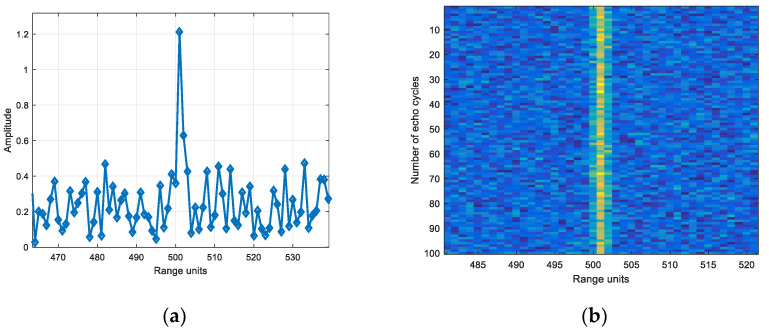
(**a**) Single frame and (**b**) 100 cycles echo diagrams with SNR of 12 dB in narrowband.

**Figure 30:**
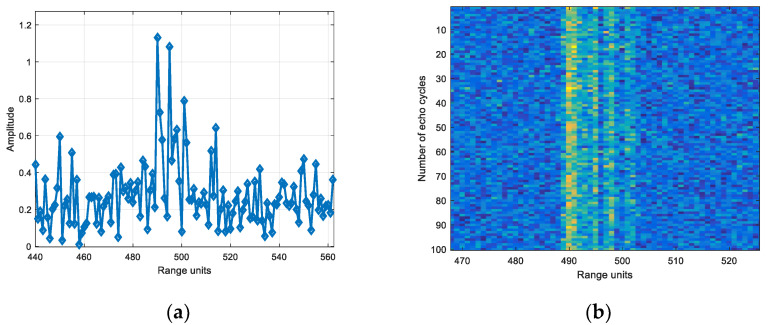
(**a**) Single frame and (**b**) 100 cycles echo diagrams with SNR of 12 dB in wideband.

Based on the echo amplitude in narrow band, the SNR was set and the white noise was added. Wideband added noise of the same energy as narrowband. Figure 29 is the echo diagram with SNR of 12 dB in narrowband, and Figure 30 is the echo diagram with SNR of 12 dB in wideband.

Comparing Figure 29a and Figure 30a, it can be seen that the target echo amplitude in narrowband is larger than the scattering point echo maximum amplitude in wideband.

**Figure 31:**
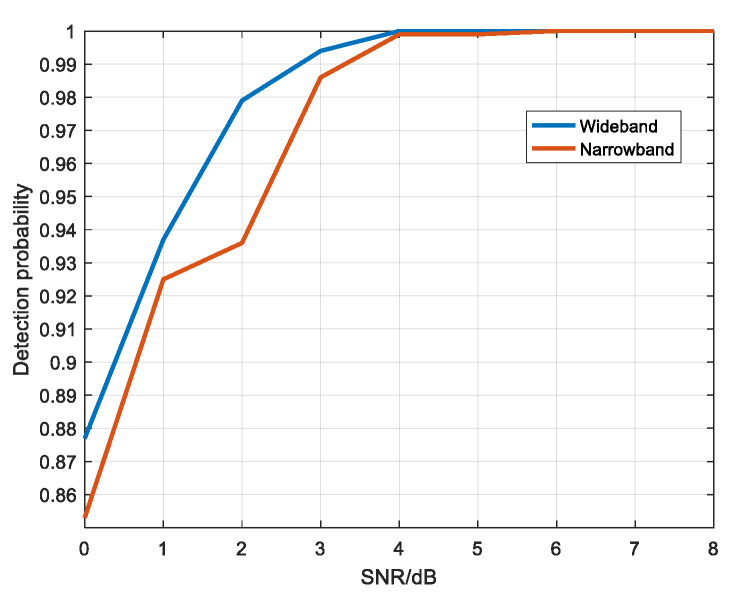
Detection probability diagram of wideband and narrowband under different SNR.

Based on the target echo amplitude in narrowband, white noise was added to make the SNR from 0 dB to 8 dB. Wideband added noise of the same energy as narrowband. The wideband and narrowband multi-period echo diagrams were divided into 20 × 20 image blocks and detected by convolutional neural network. Figure 31 shows the detection probability diagram of wideband and narrowband under different SNR conditions.

As can be seen from the figure, although the amplitude value of a single scattering point in wideband is smaller than that of the target echo in narrowband, the detection probability of wideband is slightly higher than that of narrowband when the SNR is low.

#### 4.3.2. Electromagnetic Simulation Experiment II

**Figure 32:**
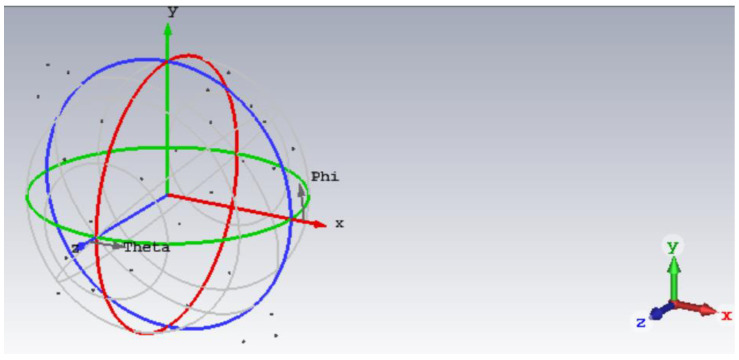
Electromagnetic simulation experiment II model diagram.

The X-axis of the model in Section 4.3.1 was rotated by 5°, the model is shown in Figure 32. Electromagnetic simulation was carried out for the echoes in the case of wideband and narrow band. The frequency is [9.5, 10.5] GHz in the case of wideband and [9.995, 10.005] GHz in the case of narrowband.

**Figure 33:**
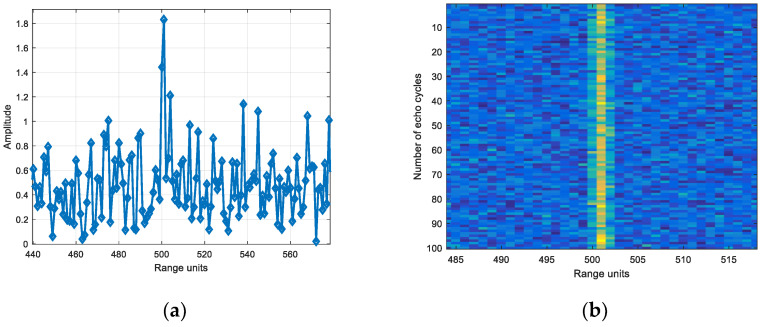
(**a**) Single frame and (**b**) 100 cycles echo diagrams with SNR of 12 dB in narrowband.

**Figure 34:**
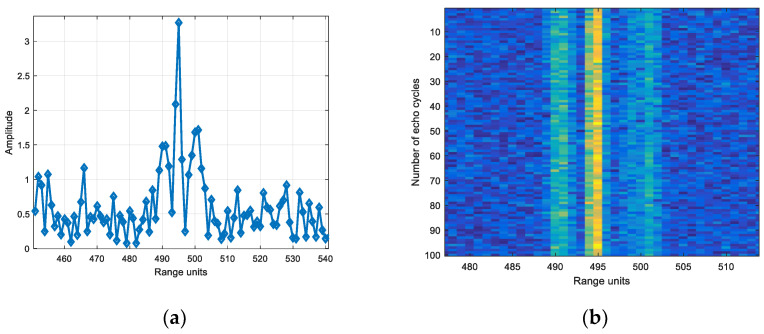
(**a**) Single frame and (**b**) 100 cycles echo diagrams with SNR of 12 dB in wideband.

Based on the echo amplitude in narrow band, the SNR was set and the white noise was added. Wideband added noise of the same energy as narrowband. Figure 33 is the echo diagram with SNR of 12 dB in narrowband, and Figure 34 is the echo diagram with SNR of 12 dB in wideband.

Comparing Figure 33a and Figure 34a, it can be seen that the target echo amplitude in narrowband is smaller than the scattering point echo maximum amplitude in wideband.

**Figure 35:**
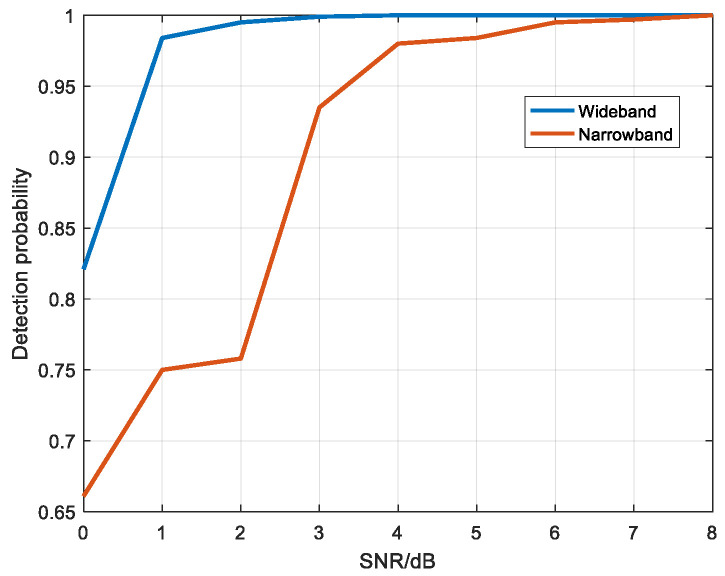
Detection probability diagram of wideband and narrowband under different SNR.

Based on the target echo amplitude in narrowband, white noise was added to make the SNR from 0 dB to 8 dB. Wideband added noise of the same energy as narrowband. The wideband and narrowband multi-period echo diagrams were divided into 20 × 20 image blocks and detected by convolutional neural network. Figure 35 shows the detection probability diagram of wideband and narrowband under different SNR conditions.

As can be seen from the Figure 35, when the amplitude value of the narrowband target echo is smaller than the maximum amplitude value of the wideband scattering point echo, the detection probability is lower than that of wideband and the detection effect is worse than that of wideband when the SNR is low.

Through two electromagnetic simulation experiments, it can be verified that the proposed method has a good detection effect on the target under the condition of low SNR.

## 5. Conclusions

In this paper, a radar target signal detection method based on convolutional neural network for graphically expressed radar signals is proposed to solve the problem of low probability of radar target signal detection under the condition of low signal-to-noise ratio. Considering the requirements of convolutional neural network input expression form, the target echo signal is processed graphically, and the network is designed and optimized. 

Through the simulation experiment in this paper, the following conclusions can be obtained: Firstly, convolutional neural network can be used for radar target signal detection, and the detection is effective. By comparing with the multi-pulse accumulation detection method, the effectiveness of the proposed method for the detection of single scatterer with low signal-to-noise is verified: in the case of −5 dB signal-to-noise, the detection probability of the proposed method is higher than 0.9, while the detection probability of the multi-pulse accumulation detection method is lower than 0.24. Secondly, the effect of the proposed method is different from that of multi-period pulse accumulation gain. By inspecting image blocks with different numbers of cycles and comparing them with the multi-period accumulation method, it was proved that the effect gain of convolutional neural network detection method is not linearly related to the number of cycles of image blocks. Thirdly, convolutional neural network can be used to detect various target signals under certain conditions. Through the detection simulation experiments of moving scatterers, two scatterers in different relative positions and multiple scatterers, the effectiveness of convolutional neural network is verified.

Convolutional neural network does have some good effects in radar target signal detection, but there is some lack of interpretation for the physical significance of convolutional neural network detection. The mechanism of convolutional neural network for target signal detection will be further studied in the future.

## Figures and Tables

**Table 1:** The parameters of the convolutional neural network in this paper.

Layer Name	Parameter Settings
Input	n×n
Conv2D	Filters = 6 kernel_size = 5 × 5
BN layer	batch_size = 32
Activation layer	Activation = ‘sigmoid’
Maxpool2D	pool_size = 2 × 2 strides = 2
Conv2D	Filters = 16 kernel_size = 5 × 5
BN layer	batch_size = 32
Activation layer	Activation = ‘sigmoid’
Maxpool2D	pool_size = 2 × 2 strides = 2
Dense(120)	units = 120 activation = ‘sigmoid’
Dense(84)	units = 84 activation = ‘sigmoid’
Softmax	units = 2 activation = ‘softmax’

**Table 2:** The simulation parameters of LFM.

Parameters	Symbols	Value
Carrier frequency	$f_{0}$	10 GHz
Bandwidth	B	1 GHz
Sub-pulse width	$T_{p}$	100 μs
Sampling rate	$f_{s}$	2 GHz

**Table 3:** The power values of 20 echo period signals under the condition of 0 dB SNR.

	Signal Power/dB	Noise Power/dB	SNR/dB
1	25.3	12.7	12.6
2	27.3	12.7	14.6
3	25.7	12.7	13.0
4	26.2	12.7	13.5
5	23.6	12.7	10.9
6	24.5	12.7	11.8
7	28.6	12.8	15.8
8	25.6	12.7	12.9

**Table 4:** The coordinate positions of 27 metallic spheres.

Serial Number	Coordinate Position/cm	Serial Number	Coordinate Position/cm	Serial Number	Coordinate Position/cm
1	[0, 0]	10	[116.7, 92.5]	19	[92.5, 116.7]
2	[26.2, 7.3]	11	[141.1, 118.7]	20	[66.3, 92.4]
3	[55.5, 27.0]	12	[162.8, 117.7]	21	[39.1, 127.4]
4	[95.5, 9.8]	13	[177.4, 133.5]	22	[16.1, 156.8]
5	[135.5, −7.4]	14	[157.7, 148.6]	23	[1.5, 159.3]
6	[159.3, 1.5]	15	[148.6, 157.7]	24	[−7.4, 135.5]
7	[156.8, 16.1]	16	[133.5, 177.4]	25	[9.8, 95.5]
8	[127.4, 39.1]	17	[117.7, 162.8]	26	[27.0, 55.5]
9	[92.4, 66.3]	18	[118.7, 141.1]	27	[7.3, 26.2]

## Data Availability

The data presented in this study are available on request from the corresponding author.
